# Sequelae of Acute Pulmonary Embolism: From Post-Pulmonary Embolism Functional Impairment to Chronic Thromboembolic Disease

**DOI:** 10.3390/jcm13216510

**Published:** 2024-10-30

**Authors:** John H. Fountain, Tyler J. Peck, David Furfaro

**Affiliations:** 1 Division of Pulmonary, Critical Care and Sleep Medicine, Beth Israel Deaconess Medical Center, Boston, MA 02215, USA; jfounta2@bidmc.harvard.edu (J.H.F.); dfurfaro@bidmc.harvard.edu (D.F.); 2 Harvard Medical School, Boston, MA 02115, USA

**Keywords:** pulmonary embolism, pulmonary hypertension, CTED, CTEPH, post-pulmonary embolism syndrome, quality of life

## Abstract

Among survivors of acute pulmonary embolism (PE), roughly half report persistent dyspnea, impaired functional status, and decreased quality of life. Post-pulmonary embolism syndrome (PPES) is a broad condition which has been increasingly recognized in recent years and may be due to post-pulmonary embolism functional impairment, chronic thromboembolic disease, or the most severe long-term complication of PE, chronic thromboembolic pulmonary hypertension. Despite guideline recommendations for appropriate follow-up for post-pulmonary embolism patients, PPES remains underrecognized and diagnostic testing underutilized. Patients with symptoms suggestive of PPES at follow-up should undergo a transthoracic echocardiogram to screen for the presence of pulmonary hypertension; additional testing, such as a ventilation/perfusion scan, right heart catheterization, and cardiopulmonary exercise testing may be indicated. The pathophysiology of post-pulmonary embolism syndrome is complex and heterogeneous. In chronic thromboembolic pulmonary hypertension, the pathophysiology reflects persistent pulmonary arterial thrombi and a progressive small vessel vasculopathy. In patients with chronic thromboembolic disease or chronic thromboembolic pulmonary hypertension, medical therapy, balloon pulmonary angioplasty, or pulmonary thromboendarterectomy should be considered, and in cases of chronic thromboembolic pulmonary hypertension, pulmonary thromboendarterectomy significantly improves mortality. In all causes of post-pulmonary embolism syndrome, rehabilitation is a safe treatment option that may improve quality of life.

## 1. Introduction

Post-pulmonary embolism syndrome (PPES) is a broad and heterogeneous condition that has been increasingly recognized after acute pulmonary embolism (PE). Despite advances in the detection and management of acute PE, roughly half of patients report dyspnea, exercise intolerance, impaired functional status, or decreased quality of life at follow-up [1,2,3,4]. While complications of PE, such as recurrent venous thromboembolism (VTE) and anticoagulant-related bleeding, have been well-described, PPES remains under-recognized and undertreated.

## 2. Definition and Epidemiology

PPES is heterogeneous and is described as one of the following syndromes despite at least three months of anticoagulation after acute PE: post-PE functional impairment, chronic thromboembolic pulmonary disease (CTED), or chronic thromboembolic pulmonary hypertension (CTEPH) (Table 1) [1]. Post-PE functional impairment—defined as the presence of dyspnea, impaired exercise tolerance, or diminished functional status without an identified non-PE explanation, CTED, or CTEPH—represents the most common form of PPES.

The symptoms of PPES are non-specific and thus it remains under-recognized and diagnostic testing is under-utilized. In a retrospective cohort study of 21,297 patients with their first PE, PPES was present in 56.2% of patients at follow-up; however, only 42.8% of these patients had an appropriate diagnostic testing ordered [5]. While the high incidence of PPES after PE is replicated in multiple patient cohorts, the risk factors have not been fully elucidated and vary across the spectrum of PPES [1,3,4,10,11,12]. Cardiopulmonary comorbidities, age, higher BMI, and smoking are predictive of post-PE functional impairment [2]. Several risk factors for the development of CTEPH have been identified, which include prior VTE, malignancy, the presence of antiphospholipid antibodies, history of splenectomy, chronic inflammatory disease, ventriculoatrial shunts, and hypothyroidism [8,13,14].

While there has been great interest in therapies for acute PE to prevent decompensation and mortality, these treatments have yet to demonstrate prevention of PPES or CTEPH. Among 109 previously healthy patients with submassive PE treated with anticoagulation alone, the cornerstone of PE therapy, 41% had abnormal cardiopulmonary function at a 6 month follow up, characterized by an abnormal right ventricular size and/or function, an impaired 6-min walk distance, and/or a NYHA functional class > II [4]. Notably, in a long-term follow up of the PEITHO trial—which evaluated fibrinolysis for intermediate-risk PE—there was no difference in the proportion of patients who had CTEPH or post-PE impairment between the anticoagulation and tenecteplase treatment arms [15]. Further, to date, there is no evidence that mechanical thrombectomy or catheter-directed therapies reduce the risk of PPES.

Importantly, reported symptoms of PPES correlate well with objective findings of exercise performance. The ELOPE study evaluated patients after their first PE and performed quality of life (QoL) questionnaires, a 6-min walk test (6MWT), and a cardiopulmonary exercise test (CPET) at 1 and 12 months, as well as a ventilation/perfusion (V/Q) scan and computed tomography pulmonary angiography (CT-PA) at 6 and 12 months. Patients with a VO2 peak < 80% predicted had worse generic and PE-specific QoL scores, dyspnea scores, and 6-min walk distances (6MWD) [3]. While these relationships are well replicated, the significance of other findings, such as residual pulmonary vascular obstruction, remains less clear. In the ELOPE study, the mean obstruction index on CT-PA was similar between patients with reduced and normal VO2 peaks, suggesting that both initial and residual obstruction does not directly correlate to symptoms after PE [3]. In contrast, other studies have found that thrombus resolution is associated with improved NYHA functional class and thus the presence of residual obstruction may represent a risk factor for PPES [11].

The timely diagnosis of PPES is crucial, particularly to identify patients with CTEPH given its mortality if left untreated and the multiple available treatment modalities, including curative surgical options. The incidence of CTEPH has been estimated from 0.79–3.8% after PE, and a recent meta-analysis estimates its incidence to be 3.2% in survivors of PE and 0.56% in all-comers after PE [8,12,13,16,17,18]. Further making the diagnosis of PPES more challenging, up to 25% of patients with CTEPH have no known history of PE [19].

## 3. Pathophysiology

### 3.1. Post-PE Functional Impairment

Of the etiologies of PPES, post-PE functional impairment remains the least well understood. It is hypothesized to be due to deconditioning secondary to dyspnea from PE, persistent chest pain, or fear of complications or recurrence of PE [1]. Many survivors of PE report mental health conditions that contribute to functional impairment including depression, anxiety, and post-traumatic stress [20,21]. It remains a diagnosis of exclusion in symptomatic patients who have been confirmed not to have CTED or CTEPH. Further data, such as tissue pathology from biopsy or autopsy, may help elucidate its etiology.

### 3.2. CTED and CTEPH

**Figure 1:**
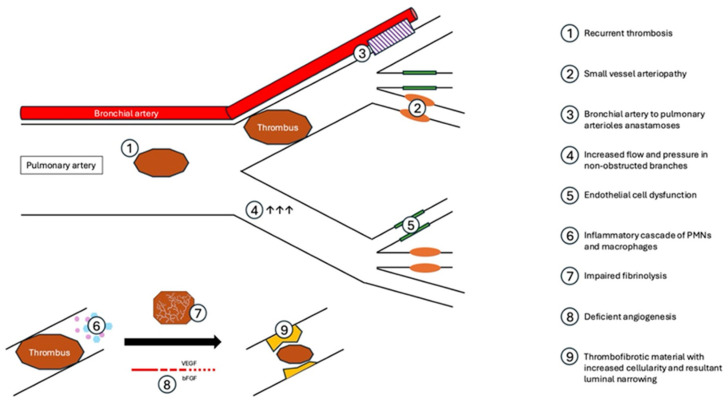
Pathophysiology of CTEPH.

Patients with CTED and CTEPH demonstrate residual obstruction in the pulmonary vasculature, though the fact that only some patients develop pulmonary hypertension indicates a complex physiologic pathway beyond just organization of chronic thrombus (Figure 1). Patients with PE are well known to be at high risk of recurrent VTE, with 10.1% of patients having recurrent VTE at 6 months and 40% at 10 years [22]. Further, patients with CTEPH demonstrate increased platelet activation and have higher rates of lupus anticoagulant, antiphospholipid antibodies, and factor V Leiden compared to patients with non-CTEPH pulmonary hypertension [23,24]. Additionally, fibrin resistance to plasmin-mediated lysis has been observed in CTEPH patients, suggesting a role of impaired fibrinolysis in the development of persistent luminal abnormalities following PE in this population [25]. As such, thorough evaluations for hypercoagulable disorders and recurrent VTE are essential in the evaluation of CTED and CTEPH.

Invasive hemodynamic and imaging studies have shown that >25% of the cumulative pulmonary arterial lumen must be obstructed in acute PE prior to the PA pressure rising, which suggests that vascular changes occur in areas beyond those with unresolved thrombus [2]. Histopathology from surgical specimens after PTE provide insight into these progressive vascular changes, and demonstrate small vessel arteriopathy [26], with findings historically characteristic of pulmonary arterial hypertension (PAH): plexogenic lesions, smooth muscle hypertrophy, and intimal proliferation and fibrosis [26,27]. Notably, these changes can be seen both in vessels distal to PE and those free of thrombus, and, compared to patients with PAH, they generally occur in larger caliber vessels in CTEPH and are more heterogeneous [27]. The presence of arteriopathy distal to obstructed vessels may be due to the development of bronchial-to-pulmonary vascular anastomoses, pulmonary arterial remodeling, and abnormal vascular reactivity with related endothelial cell dysfunction [24,27]. The cause of remodeling in non-obstructed vessels is not fully understood but may be related to high flow rates and higher-pressure circulation in these areas [28].

After acute PE, there is a rapid inflammatory response consisting of inflammatory cells, cytokines, and chemokines [29]. In a rat model of PE, there was a significant increase in neutrophil and macrophage concentration within the vasa vasorum at 1 and 2 days after PE with increased intimal wall thickness at 4 days and increased cellularity through 14 days [29]. Impairments within the usual process of organization and degradation of thrombus, recanalization and remodeling of the vascular wall may lead to excessive remodeling [24]. Resultant CTEPH may also be secondary to deficient angiogenesis, in which abnormalities in neovascularization, mediated by vascular endothelial growth factor (VEGF) and basic fibroblast growth factor (bFGF), lead to impaired penetration of an occlusive thrombus and impaired recanalization [24].

## 4. Detection and Imaging Findings

The timely identification of PPES is paramount, particularly to identify CTEPH. Despite increases in the awareness of PPES, and particularly CTEPH, the median time from the development of symptoms to diagnosis was 14.1 months in a registry of 679 patients with CTEPH [19]. As a result, guidelines for the diagnosis and management of acute PE recommend transthoracic echocardiogram (TTE) at 3–6 months after PE if dyspnea or functional limitation persist despite anticoagulation [30]. Acknowledging the practical limitations of obtaining TTE in some settings, the InShape II trial evaluated an algorithm utilizing clinical characteristics, electrocardiogram, and biomarkers to identify patients who were unlikely to have CTEPH, and thus did not warrant TTE. Of 424 patients with acute PE, 81 (19%) were referred to TTE based on their algorithm, and only 1 out of 343 (0.29%) patients who were deemed low risk was subsequently found to have CTEPH [31]. This may represent a viable option for screening in resource-limited settings.

Patients with intermediate- or high-probability of pulmonary hypertension on TTE should undergo further diagnostic evaluation for persistent vascular obstruction in order to diagnose or exonerate CTEPH. V/Q scan is the first line imaging modality for diagnosing CTED and has a sensitivity of 96–97% and specificity of 90–95% [30]. While CT-PA remains an excellent test for the diagnosis of acute PE, it is not recommended in isolation for evaluation of CTEPH; among a cohort of patients with CTEPH who underwent both a V/Q scan and a CT-PA, the sensitivity of the V/Q scan was 98.9% compared to 65.9% for the CT-PA [32]. Nonetheless, imaging findings on the CT-PAs that are suggestive of CTEPH are helpful when present, and include the presence of persistent thrombus, eccentric wall-adherent thrombus, pulmonary arterial webs, abrupt tapering of the pulmonary arteries, bronchial artery collaterals, and a widening of the main pulmonary artery [33]. Other imaging modalities, such as single-photon emission computed tomography (SPECT) [34], dual-energy CT, and magnetic resonance (MR) pulmonary angiography, can also demonstrate perfusion defects and may play a role in CTEPH diagnosis in the future, especially given the increased resolution that these studies provide [35].

Cardiopulmonary exercise testing (CPET) remains a useful diagnostic tool for those with dyspnea after PE and may be diagnostic or highly suggestive of CTED or CTEPH based on characteristic physiologic abnormalities. Patients with post-PE functional impairment without CTED/CTEPH generally have a reduction in peak VO2, which supports deconditioning as its cause. In patients with CTED and CTEPH, however, CPET demonstrates an increase in ventilatory dead space proportion (VD/VT), which corresponds to areas of impaired perfusion due to persistent thrombus [36]. Held et al. evaluated the use of a 4- and 6-score parameter test using CPET in patients with CTEPH that had normal or unmeasurable right ventricular systolic pressure on a TTE [37]. Their 4-score parameter testing evaluated increases in the alveolar–arterial oxygen gradient, minute ventilation to carbon dioxide production (VE/VCO_2_) slope, capillary to end-tidal carbon dioxide gradient (P[c-ET]CO_2_), and end-tidal partial pressure of CO_2_ at anaerobic threshold (PETCO_2_ at AT), and was 83.3% sensitive and 92.2% specific for CTEPH [37].

Ultimately, right heart catheterization (RHC) is the gold standard for diagnosis of pulmonary hypertension and is required to definitively diagnose CTEPH. CTEPH is confirmed when patients have persistent perfusion abnormalities after effective anticoagulation and the presence of pre-capillary pulmonary hypertension on RHC. Pre-capillary pulmonary hypertension is defined as PA pressure ≥ 20 mmHg with pulmonary capillary wedge pressure ≤ 15 mmHg and PVR ≥ 2 woods units [9]. Concomitant pulmonary angiography is helpful to confirm vascular obstruction and determine whether patients are a candidate for surgical or interventional management. All patients with confirmed CTEPH should be tested for anti-phospholipid syndrome, and hypercoagulability work up in patients with PPES should otherwise be considered based on patient risk factors, clotting history, and family history.

## 5. Management

Guidelines recommend the referral of CTEPH patients to a CTEPH expert center once diagnosed for consideration of surgical/interventional treatment options, medical therapy, and multimodal therapy [9,38,39].

### 5.1. Anticoagulation

The backbone of therapy for patients with CTEPH is indefinite anticoagulation (Table 2). For patients with post-PE functional impairment and CTED without PH, the duration of anticoagulation should be determined based on guideline recommendations following acute PE and patient-level decision making; the nuances of anticoagulant choice and duration after PE are beyond the scope of this review [30,40,41]. For patients with CTEPH, vitamin K antagonists (i.e., warfarin) are the preferred anticoagulants based on retrospective data, suggesting that patients on direct oral anticoagulants have increased rates of recurrent VTE with similar bleeding rates [42,43]. As patients with prior VTE are at risk of recurrent thrombosis, they should be monitored for the presence of new thrombotic events at follow up.

### 5.2. Pulmonary Thromboendarterectomy

Pulmonary thromboendarterectomy (PTE) has become a well-established procedure that is performed on patients with CTED and CTEPH. The technique of PTE is well described in the literature and a few key points warrant mentioning. An effective PTE is by definition bilateral, and thus requires a midline sternotomy. A cardiopulmonary bypass and a brief circulatory arrest are necessary in contributing to the risk profile of the procedure. An endarterectomy is performed by dissection into the pulmonary vasculature as well as a removal of pathologically remodeled tissue in addition to organized thrombus; this is performed on the subsegmental branches [44,45].

All patients with CTEPH should have multidisciplinary evaluation for PTE, as it represents a potentially curative treatment. Surgical candidacy depends on the anatomy of the disease (proximal vs. distal), the severity of the pulmonary hypertension, the risk profile of the patient, and the experience level of the treating center [9,45]. Left untreated, CTEPH leads to progressive RV failure and death, and in one cohort, the untreated mortality at 3 years was 90% in patients with a mean PA pressure of >30 mm Hg [46]. To that end, multiple studies have evaluated the efficacy and safety of PTE for CTEPH. In a Canadian study of 401 patients who underwent PTE, the 30-day mortality was 2.8% and the 5-year survival rate was 80–91% depending on disease type [47]. These patients demonstrated a significant improvement in 6MWD, right ventricular systolic pressure, and PVR, and a significant reduction in NYHA functional class. Patients with severe pulmonary hypertension (based on PVR >1000 dynes/s/cm^−5^) had a higher need for ECMO, and a longer duration of intubation, ICU, and hospital stay but had a similar 30-day mortality rate to patients with PVR < 1000 dynes/s/cm^−5^ and a 10-year survival of 84% [48]. Among >1500 patients treated at University of California San Diego, in-hospital mortality decreased temporally and with increased experience from 5.5% to 2.2%, and cumulative survival at 5 years was 92% [45]. Notably, this cohort included a large proportion of patients with distal disease indicating that with appropriate experience even distal chronic perfusion defects can be treated effectively and definitively with PTE [45]. Beyond survival, both echocardiographic and CPET parameters show improvement after PTE; sustained improvements in RV size, right ventricular systolic pressure, and TR at 1-year post-surgery [49] and improvements in peak VO2 and reduction in VE/VCO_2_ slope over the first year after PTE [50] have been demonstrated.

Historically, PTE was indicated only for patients with CTEPH; however, recent data has supported its use in patients with CTED. A 2014 study performed PTE in patients with CTED with a concomitant IVC filter placement and demonstrated a reduction in mean PA pressure from 21 to 18 mm Hg, an increase in 6MWD from 372 to 421m, a reduction in PVR from 164 to 128 dynes/s/cm^−5^, and an improvement in NYHA functional class [51]. All patients were alive at hospital discharge, though two died after discharge, resulting in a mortality of 5%. A 2018 study of 23 patients showed similar results and survival characteristics [52]. Of note, given the changes in the ERS/ESC diagnostic criteria for pulmonary hypertension, was that many of the patients in these studies would meet the criteria for CTEPH by the updated definition, so the utility of PTE in CTED requires further investigation.

### 5.3. Balloon Pulmonary Angioplasty

Balloon pulmonary angioplasty (BPA) is a treatment option for patients with inoperable CTEPH or persistent or recurrent pulmonary hypertension after PTE, and there have been multiple studies evaluating its safety and efficacy. BPA is performed endovascularly, primarily in the catheterization laboratory, with advancement of a catheter to chronic thromboembolic lesions and inflation of a balloon causing disruption to intimal caps, the dilation of fibrotic luminal obstructions, the compression of organized thrombotic material, and the stretching of the pulmonary vessels [53,54]. In a Japanese cohort of 308 patients, the average 6MWD increased from 318 to 429.7m with an improvement in the b-type natriuretic peptide (BNP) from 239.5 to 38.7 pg/mL, PVR from 853.7 to 288.1 dynes/s/cm^−5^, and mean PA pressure from 43.2 to 22.5 mm Hg [55]. Average NYHA functional class improved from III to II. Mortality was 3.9% at follow up, with eight patients (2.6%) dying within 30 days of BPA. French [56], German [57], and American [58] registries demonstrate similar improvements in hemodynamics and 6MWD after BPA as well as low mortality in the peri-procedural period (2.2%, 1.8%, and 1.3%, respectively) with patients undergoing an average of 5.4, 4.8, and 2.7 sessions, respectively.

As clinicians may offer procedural therapy and/or medical therapy to patients with CTEPH, trials have sought to elucidate their efficacy and safety in head-to-head trials. Recently, the RACE trial evaluated 105 patients randomized to riociguat, an oral medication that both sensitizes soluble guanylate cyclase to nitric oxide and acts as a soluble guanylate cyclase agonist causing vasodilation of the pulmonary vasculature, or BPA. In patients who underwent BPA, there was an average reduction in PVR of 458.4 dynes/s/cm^−5^ compared to 200.8 in patients treated with riociguat [59]. There was no difference in 6MWD between the two groups; however, there were improvements in the borg dyspnea scale, WHO-FC, NT-proBNP, and mean PA pressure in patients who underwent BPA compared to treatment with riociguat [59]. Similar findings were noted in the MR BPA trial, which randomized patients to treatment with riociguat or BPA and demonstrated reduction in the mean PA pressure of 16.3 mm Hg in the BPA group and 9.3 mm Hg in the riociguat group at 12 months [60]. However, especially considering the frequent need for multiple sessions, the procedural risk of BPA warrants careful consideration. The most common complications across both cohorts were lung injury and hemoptysis [59,60], and unsurprisingly, an increase in BPA experience reduced the complication rate [56].

Given these, and other similar results, BPA is increasingly utilized for CTEPH, while acknowledging that PTE is the gold standard to pursue if feasible. The current ESC/ERS guidelines for the management of pulmonary hypertension recommend BPA as a class I indication for patients with residual PH after PTE or who are technically inoperable, with weaker recommendations to consider BPA for patients based on surgical risk profile alone [9].

### 5.4. Medical Therapy

Medical therapy remains a well-tolerated option for patients with CTEPH and has been evaluated in patients with residual pulmonary hypertension after PTE or BPA as well as in those with inoperable disease. The most well-studied medication for CTEPH is riociguat. In the CHEST-1 study and its subsequent follow up, CHEST-2, riociguat was shown to reduce PVR and increase 6MWD compared to a placebo, with similar rates of adverse events between the two groups [61,62].

Endothelin receptor antagonists have also been evaluated in CTEPH. The MERIT-1 trial demonstrated a reduction in PVR and increase in 6MWD with the use of macitentan compared to a placebo [63], while the BENEFIT trial demonstrated a reduction in PVR and an increase in 6MWD with the use of bosentan compared to a placebo [64].

For patients with severe disease and functional impairment, subcutaneous treprostinil has been studied in a randomized controlled trial of 105 patients and demonstrated improvement in 6MWD [65]. While other medical therapies for pulmonary hypertension are not specifically approved for CTEPH, such as phosphodiesterase type 5 inhibitors and other forms of prostacyclin analogs, registry data demonstrates that they are frequently used for monotherapy or combination therapy at the discretion of specialists [66,67].

### 5.5. Rehabilitation

Rehabilitation is a safe and low risk treatment option for all causes of PPES. Several studies have demonstrated the safety of rehabilitation after venous thromboembolism [68,69,70,71]; there were no deaths and few adverse events related to VTE in any patients in these studies. Notably, the majority of studies do not differentiate between patients with post-PE functional impairment, CTED, and CTEPH, making results difficult to generalize by disease process. Among 23 patients with PE (5 of whom had massive PE), a 3-month exercise program led to an improvement in peak VO2 by 3.9 mL/kg/min, the time to walk 400 m by 1.1 min, and an improvement in QoL questionnaires [69]. Patients enrolled in a 6-week pulmonary rehabilitation program after PE demonstrated an increase in 6MWD by 49.4 m, with 78% of patients reporting an improvement in their health status after rehabilitation [72]. In patients with PPES but without CTEPH, a study of 27 patients demonstrated an improvement in the QoL questionnaires and an improvement in post-VTE functional status with the use of a 12-week pulmonary rehabilitation program [73]. These results have been replicated for patients with confirmed CTEPH. In a study of 35 patients with inoperable or residual CTEPH, a 3-week in-hospital followed by 15-week out of hospital exercise program led to an improvement in 6MWD QoL questionnaires and peak oxygen consumption [74].

## 6. Conclusions

Post-pulmonary embolism syndrome is common, underdiagnosed, and warrants early consideration in patients with dyspnea, exercise intolerance, impaired functional status, or worse QoLs after PE. Evaluation with a TTE, a V/Q scan, and/or a CPET can identify patients who may benefit from treatment with medical, interventional, or rehabilitation-based therapy.

## Figures and Tables

**Table 1:** Definition, epidemiology, and diagnosis of the spectrum of post-pulmonary embolism syndrome (PPES).

Syndrome	Definition *	Epidemiology	Diagnostic Testing for Detection
Post-pulmonary embolism (PE) functional impairment	Dyspnea, impaired exercise tolerance, or diminished functional status after acute PE without an identified non-PE explanation, CTED, or CTEPH	Poorly reported, up to 56% of patients after acute PE have PPES [5]	6MWD, QoL questionnaire, CPET and rule out CTED or CTEPH
Chronic thromboembolic disease (CTED)	Persistent pulmonary vascular obstruction and functional limitation or symptoms without the presence of resting pulmonary hypertension	29–38% [6,7] of patients have residual perfusion defects after acute PE	Confirm persistent vascular obstruction: V/Q scan, CTPA, pulmonary angiography and rule out CTEPH with TTE and RHC
Chronic thromboembolic pulmonary hypertension (CTEPH)	Persistent pulmonary vascular obstruction and the presence of pre-capillary pulmonary hypertension on right heart catheterization	0.56% in all comers after PE [8]	Confirm persistent vascular obstruction: V/Q scan, CTPA, pulmonary angiography and RHC with mPA ≥ 20 mmHg, PCWP ≤ 15 mmHg and PVR ≥ 2 woods units [9]

* All diagnoses can only be made after 3 months of effective anticoagulation following acute PE. PPES = post-pulmonary embolism syndrome; PE = pulmonary embolism; CTED = chronic thromboembolic disease; CTEPH = chronic thromboembolic pulmonary hypertension; CPET = cardiopulmonary exercise test; V/Q = ventilation/perfusion; CTPA = computed tomography pulmonary angiogram; TTE = transthoracic echocardiogram; RHC = right heart catheterization; mPA = mean pulmonary artery pressure; PCWP = pulmonary capillary wedge pressure; PVR = pulmonary vascular resistance identified non-PE explanation; CTED or CTEPH—represents the most common form of PPES. Among patients with persistent pulmonary vascular obstruction on imaging, there are two groups of patients: those without pulmonary hypertension at rest but with functional limitation, who are described as having CTED, and those who meet criteria for CTEPH with pulmonary hypertension at rest (mean pulmonary arterial [PA] pressure ≥ 20 mmHg with pulmonary capillary wedge pressure ≤ 15 mmHg and pulmonary vascular resistance [PVR] ≥ 2 woods units [9]). CTEPH is the most severe long-term complication of PE.

**Table 2:** Treatments for post-pulmonary embolism syndrome (PPES).

Syndrome	Treatment
Post-pulmonary embolism (PE) functional impairment	Anticoagulation Duration and agent per guidelines following acute PE and patient risk factors Pulmonary rehabilitation Treatment for concomitant mental health conditions common after PE (depression, anxiety, PTSD)
Chronic thromboembolic disease (CTED)	Anticoagulation Duration and agent per guidelines following acute PE and patient risk factors Consider longer duration or lifelong in presence of significant persistent perfusion defects Pulmonary rehabilitation Consider PTE Small studies of use in patients with CTED Further studies needed
Chronic thromboembolic pulmonary hypertension (CTEPH)	Indefinite anticoagulation Vitamin K antagonists preferred over DOACs Pulmonary rehabilitation PTE Multidisciplinary discussion of candidacy for all patients Gold standard treatment for patients with surgically amenable disease and appropriate risk/benefit profile BPA For patients with inoperable CTEPH For patients with persistent or recurrent CTEPH after PTE Pulmonary vasodilators Riociguat ERAs Subcutaneous Treprostinil Off-label use of other class of pulmonary vasodilators (PDE5i, prostacyclin analogs) Consider combination therapy

PE = pulmonary embolism; PTSD = post-traumatic stress disorder; CTED = chronic thromboembolic disease; PTE = pulmonary thromboendarterectomy; DOAC = direct oral anticoagulant; CTEPH = chronic thromboembolic pulmonary hypertension; BPA = balloon pulmonary angioplasty; ERA = endothelin receptor antagonist; PDE5i = phosphodiesterase type 5 inhibitor.
